# Proliferative Diabetic Retinopathy Microenvironment Drives Microglial Polarization and Promotes Angiogenesis and Fibrosis via Cyclooxygenase-2/Prostaglandin E2 Signaling

**DOI:** 10.3390/ijms252011307

**Published:** 2024-10-21

**Authors:** Shuta Kishishita, Ayumi Usui-Ouchi, Yasuo Ouchi, Yuiko Hata, Nobuyuki Ebihara, Shintaro Nakao

**Affiliations:** 1 Department of Ophthalmology, Juntendo University Urayasu Hospital, 2-1-1 Urayasu, Chiba 279-0021, Japan; 2 Department of Regenerative Medicine, Graduate School of Medicine, Chiba University, Chiba 263-8522, Japan; 3 Department of Ophthalmology, Juntendo University Graduate school of Medicine, 2-1-1 Bunkyo, Tokyo 113-8421, Japan

**Keywords:** human microglia, diabetic retinopathy, hypoxia, inflammation, neurovascular unit, COX-2

## Abstract

Diabetic retinopathy (DR) is the leading cause of visual impairment, particularly in the proliferative form (proliferative DR [PDR]). The impact of the PDR microenvironment on microglia, which are the resident immune cells in the central nervous system, and the specific pathological changes it may induce remain unclear. This study aimed to investigate the role of microglia in the progression of PDR under hypoxic and inflammatory conditions. We performed a comprehensive gene expression analysis using human-induced pluripotent stem cell-derived microglia under different stimuli (dimethyloxalylglycine (DMOG), lipopolysaccharide (LPS), and DMOG + LPS) to mimic the hypoxic inflammatory environment characteristic of PDR. Principal component analysis revealed distinct gene expression profiles, with 76 genes synergistically upregulated under combined stimulation. Notably, prostaglandin-endoperoxide synthase 2 (encoding cyclooxygenase (COX)-2) exhibited the most pronounced increase, leading to elevated prostaglandin E2 (PGE2) levels and driving pathological angiogenesis and inflammation via the COX-2/PGE2/PGE receptor 2 signaling axis. Additionally, the upregulation of the fibrogenic genes snail family transcriptional repressor 1 and collagen type I alpha 1 chain suggested a role for microglia in fibrosis. These findings underscore the critical involvement of microglia in PDR and suggest that targeting both the angiogenic and fibrotic pathways may present new therapeutic strategies for managing this condition.

## 1. Introduction

Diabetic retinopathy (DR) is a major complication of diabetes mellitus that leads to visual impairment and blindness worldwide [1].

In recent years, anti-vascular endothelial growth factor (VEGF) therapy has become a common treatment option for diabetic macular edema (DME) and proliferative DR (PDR). However, this therapy did not benefit 30–40% of the cases that were resistant to treatment [2,3]. Thus, anti-VEGF-resistant DME, diabetic macular ischemia, diabetic retinal neurodegeneration, and fibrovascular proliferation are the unmet needs for DR treatment, necessitating new therapeutic approaches [4,5].

The neurovascular unit (NVU), which includes neurons, glial cells, and other vascular components, plays a critical role in the maintenance of retinal health. DR was originally considered a microvascular complication caused by hyperglycemia. However, recent findings have revealed that NVU dysfunction in DR is a hallmark of neurodegeneration, inflammation, vascular hyperpermeability, ischemia, and neovascularization [6]. It is evident that these components interact with each other, causing the pathology of DR to progress from non-proliferative DR to PDR [6].

Microglia are the resident immune cells of the central nervous system that are essential for maintaining synaptic function and retinal homeostasis under normal conditions [7]. Under hyperglycemic conditions caused by diabetes, microglia become reactive and produce pro-inflammatory cytokines that lead to chronic inflammation in the early stages of DR, even before diabetic microangiopathy [8,9].

Previous studies using animal models of diabetes have demonstrated significant changes in microglial activation and behavior, highlighting their role in DR progression [9,10]. These changes include increased microglial reactivity and production of inflammatory mediators, which contribute to the deterioration of the NVU and exacerbation of retinal damage [6].

In PDR, pathological microglia contribute to neovascularization, fibrosis, and neurodegeneration [10,11,12,13]. However, as no rodent model fully replicates the human presentation of DR, particularly PDR, and mouse microglia are distinct from human microglia in that they lack the expression of many disease-linked genes present in humans, the detailed molecular mechanisms remain unclear [14,15].

Hypoxia, which is defined as low concentration of oxygen, inhibits prolyl hydroxylase domain enzymes (PHDs), leading to hypoxia-inducible factor 1-alpha (HIF-1α) stabilization and activation, and HIF-1α activation upregulates the expression of proangiogenic factors and plays a crucial role in the pathophysiology of PDR, leading to retinal neovascularization and fibrovascular proliferation [16].

Under hypoxic conditions, microglia are stimulated to express higher levels of angiogenic factors, including VEGF and insulin-like growth factor [11,17]. This finding suggests that microglia-mediated neovascularization plays a significant role in the pathophysiology of hypoxia-induced neovascularization in PDR. However, the impact of the PDR microenvironment on microglial polarization and the specific pathological changes it may induce remain unclear.

Hyperglycemia induces innate immune activation via increased levels of Toll-like receptor 4 (TLR4), which is associated with the pathogenesis of DR, and the TLR4 genetic polymorphisms in humans are associated with DR risk [18]. Moreover, its ligand, lipopolysaccharide (LPS), a component of the outer membrane of Gram-negative bacteria, is associated with DR progression and neuroinflammatory disorders [19,20]. Intraocular inflammation accelerates DR progression [21,22].

This study aimed to analyze gene expression changes in human induced pluripotent stem cell (iPSC)-derived microglia in response to hypoxic and innate inflammatory stimuli and to elucidate the role of microglial polarization in PDR progression. Using both LPS and dimethyloxaloylglycine (DMOG), an inhibitor of PHD, to promote HIF-1α activation, mimicking the complex inflammatory and ischemic environment of the PDR, we aimed to investigate microglial polarization and its pathological roles. This study provides insights into the role of microglia in PDR and identifies potential therapeutic targets for managing the disease, particularly emphasizing the pathogenic and pleiotropic roles of cyclooxygenase (COX)-2, which is enormously and synergistically upregulated under LPS and DMOG co-stimulation, along with its downstream products and signaling pathways in microglia as a treatment target.

## 2. Results

### 2.1. Distinct Global Gene Expression Profiles in Induced Pluripotent Stem Cell-Derived Microglia (iMGs) Under Hypoxia and Immune Activation

**Figure 1:**
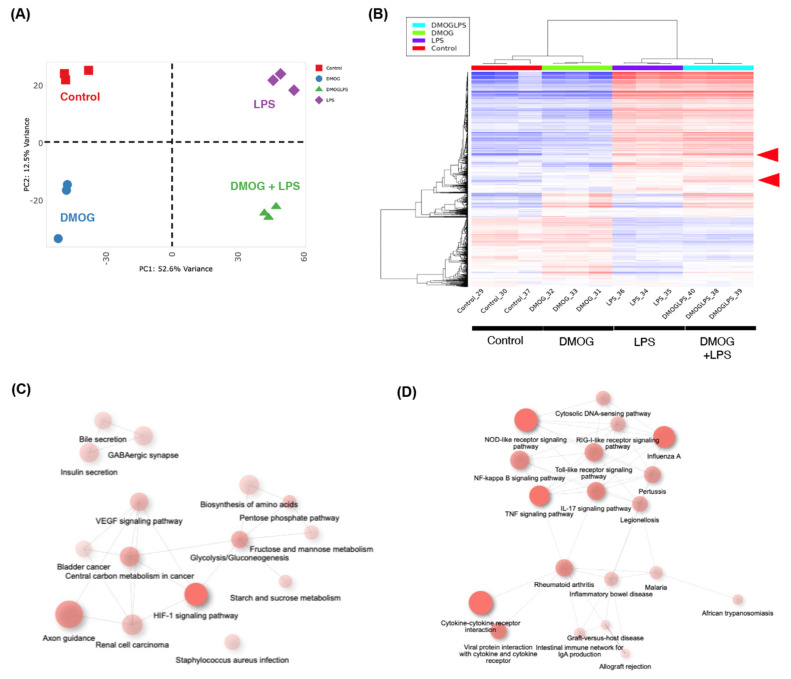
Distinct global gene expression profiles in induced pluripotent stem cell-derived microglia (iMGs) under hypoxia and immune activation. Comprehensive gene expression profiles were analyzed to assess the effects of hypoxia and innate immune activation on human iMGs using lipopolysaccharide (LPS) and dimethyloxaloylglycine (DMOG) (**A**). The heatmap shows that DMOG stimulation resulted in the significant upregulation of 216 genes, whereas LPS stimulation upregulated 912 genes compared with the control. In addition, these differences were confirmed, highlighting a subset of genes that was synergistically upregulated by both stimuli (**B**). Pathway analysis of the differentially expressed genes indicated the activation of the vascular endothelial growth factor and hypoxia-inducible factor-1 signaling pathways under DMOG stimulation, indicating a hypoxic response (**C**). Conversely, LPS stimulation enhanced Toll-like receptor signaling, nuclear factor-kappaB signaling, and tumor necrosis factor signaling, suggesting heightened immune activation (**D**).

First, we explored the comprehensive gene expression profiles to assess the effects of hypoxia and innate immune activation on human iPSC-derived microglia (iMGs). Principal component analysis revealed distinct gene expression profiles in the iMG under different stimulation conditions (LPS, DMOG, and DMOG + LPS) (Figure 1A). Specifically, DMOG stimulation resulted in the significant upregulation of 216 genes, whereas LPS stimulation upregulated 912 genes compared with the control (Figure 1B). Heatmap analysis further confirmed these differences and highlighted a subset of genes that were synergistically upregulated by both stimuli (Figure 1B). Pathway analysis of the differentially expressed genes indicated the activation of the VEGF and HIF-1 signaling pathways under DMOG stimulation, indicating a hypoxic response (Figure 1C). Conversely, LPS stimulation enhanced Toll-like receptor, nuclear factor (NF)-kappaB, and tumor necrosis factor signaling, suggesting heightened immune activation (Figure 1D). These results demonstrate that iMGs respond distinctly to LPS, DMOG, and DMOG + LPS stimulation, leading to significant alterations in their global gene expression patterns.

### 2.2. Synergistic Upregulation of Prostaglandin-Endoperoxide Synthase 2 and Pathological Gene Networks in iMGs Under Hypoxia and Immune Activation

**Figure 2:**
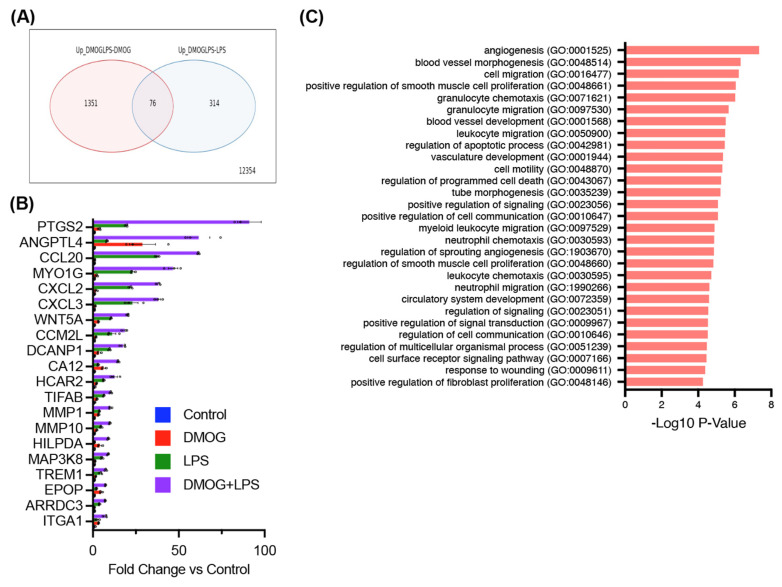
Synergistic upregulation of prostaglandin-endoperoxide synthase 2 (PTGS2) and pathological gene networks in induced pluripotent stem cell-derived microglia under hypoxia and immune activation. We found 76 genes that were significantly and synergistically upregulated by both innate immune-activating stimulation by lipopolysaccharide and hypoxic stimulation by dimethyloxaloylglycine compared with either stimulus alone (false discovery rate < 0.1, fold change > 1.5) (**A**). The top 20 synergistically upregulated genes are highlighted; the most significantly upregulated gene is PTGS2, which encodes cyclooxygenase 2 (**B**). Gene ontology analysis of the synergistically upregulated genes identified significant terms related to angiogenesis, cell migration, chemotaxis, and fibrosis (**C**).

Among the genes upregulated by both DMOG and LPS stimulation, 76 were significantly upregulated compared with either stimulus alone (false discovery rate < 0.1, fold change > 1.5) (Figure 2A). The top 20 synergistically upregulated genes are highlighted in Figure 2B, with PTGS2 encoding COX-2 showing the most significant upregulation (Figure 2B).

Gene Ontology (GO) analysis of the synergistically upregulated genes revealed significant terms related to angiogenesis, cell migration, chemotaxis, and fibrosis (Figure 2C). These findings suggest that under neuroinflammatory conditions in a hypoxic environment, microglia exhibit amplified gene expression patterns associated with pathological processes, such as angiogenesis, cell migration, chemotaxis, and fibrosis, with PTGS2 being the most markedly amplified gene among them.

### 2.3. Synergistic Upregulation of Cyclooxygenase (COX)-2 and Prostaglandin E2 (PGE2) in Response to Hypoxia and Inflammation

COX-2 is a critical mediator of inflammation and is responsible for the production of prostanoids, such as prostaglandins, in both acute and chronic inflammatory conditions [23]. COX-2 also plays a significant role in pathological angiogenesis [24,25]. To confirm the synergistic upregulation of COX-2 and subsequent prostaglandin production, we performed quantitative polymerase chain reaction (qPCR) for PTGS2 and enzyme-linked immunosorbent assay (ELISA) for prostaglandin E2 (PGE2) and prostaglandin F2α (PGF2α) under DMOG, LPS, or DMOG + LPS stimulation.

**Figure 3:**
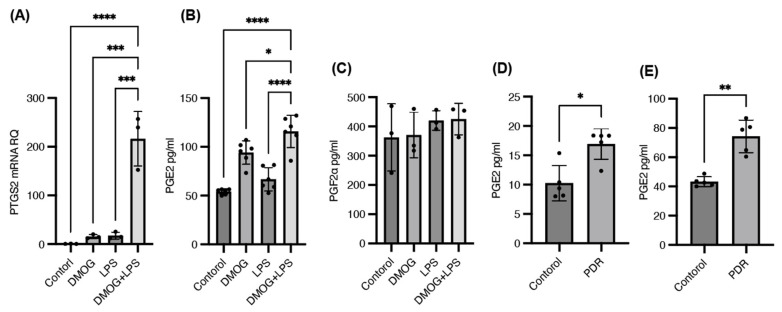
Synergistic upregulation of cyclooxygenase 2 and prostaglandin E2 in response to hypoxia and inflammation. Quantitative polymerase chain reaction for prostaglandin-endoperoxide synthase 2 (PTGS2) and enzyme-linked immunosorbent assay (ELISA) for prostaglandin E2 (PGE2) and prostaglandin F2α (PGF2α) were performed under 200 μM dimethyloxaloylglycine (DMOG), 100 ng/mL lipopolysaccharide (LPS), or combined DMOG and LPS stimulation for 24 h. The expression of PTGS2 mRNA was significantly upregulated, showing a 10–20-fold increase with single LPS or DMOG stimulation and a 150–200-fold increase with combined DMOG and LPS stimulation compared with the control (**A**). ELISA measurements indicated increased levels of PGE2, but not PGF2α, in the culture supernatant under these conditions (**B**,**C**). Aqueous humor samples were collected from five patients with proliferative diabetic retinopathy (PDR) accompanied with diabetic macular edema (two males and three females; average age, 64.4 ± 6.2 years) and five age-matched patients without any retinal diseases as control (two males and three females; average age, 64.0 ± 10.1 years) for ELISA assay. Vitreous humor samples were also collected from five patients with PDR (four males and one female; average age, 57.6 ± 5.9 years) and five age-matched patients without any retinal diseases as control (three males and two females; average age, 61.0 ± 5.2 years). PGE2 concentrations were significantly elevated in the aqueous and vitreous humors of patients with PDR compared with those in the controls (**D**,**E**). * *p* < 0.05, ** *p* < 0.01, *** *p* < 0.001, **** *p* < 0.0001.

The expression of PTGS2 mRNA was significantly upregulated, showing a 10–20-fold increase with single LPS or DMOG stimulation and a 150–200-fold increase with combined DMOG and LPS stimulation compared with the control (Figure 3A). ELISA measurements indicated increased levels of PGE2, but not PGF2α, in the culture supernatant under these conditions (Figure 3B,C). Furthermore, PGE2 concentrations were significantly elevated in the aqueous and vitreous humors of patients with PDR compared with those in controls (Figure 3D,E). PGF2α was not detected in any samples. These findings suggest that microglia activated by both hypoxia and inflammation synergistically produce PGE2 by upregulating COX-2 mRNA. This might have led to the pathological effects observed in PDR.

### 2.4. Synergistic Activation of Angiogenesis and Fibrosis-Related Genes in iMGs Under Hypoxia and Inflammation

**Figure 4:**
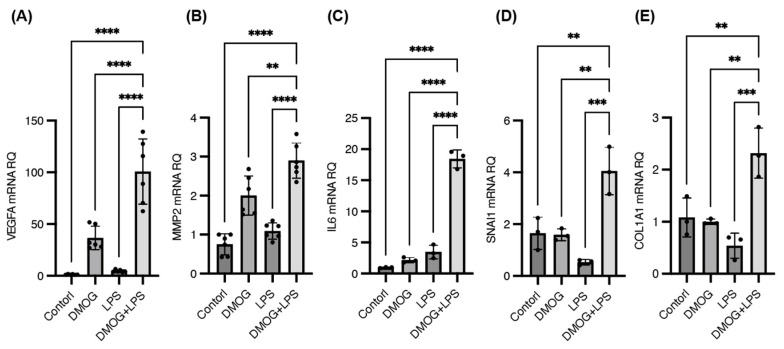
Synergistic activation of angiogenesis- and fibrosis-related genes in induced pluripotent stem cell-derived microglia under hypoxia and inflammation. Quantitative polymerase chain reaction for genes associated with angiogenesis and fibrovascular proliferation in proliferative diabetic retinopathy (PDR) were performed under 200 μM dimethyloxaloylglycine (DMOG), 100 ng/mL lipopolysaccharide (LPS), or combined DMOG and LPS stimulation for 24 h. The expression levels of vascular endothelial growth factor A, matrix metalloproteinase 2, and interleukin-6, key contributors to pathological neovascularization in PDR, were significantly upregulated under combined DMOG and LPS stimulation compared with monostimulation (**A**–**C**). Snail family transcriptional repressor 1 (SNAI1), a master transcription factor that regulates mesenchymal transition, was upregulated under both DMOG and LPS stimulation (**D**). Collagen type I alpha 1 chain, a major extracellular matrix component induced by SNAI1 activation, markedly increased under combined stimulation (**E**). ** *p* < 0.01, *** *p* < 0.001, **** *p* < 0.0001.

We investigated the expression of genes associated with angiogenesis and fibrovascular proliferation in PDR, as prompted by the results of the GO analysis, which indicated the involvement of these pathways. The expression levels of the proangiogenic factor VEGFA, matrix metalloproteinase 2 (MMP2), and the inflammatory cytokine interleukin-6 (IL-6), which are key contributors to pathological neovascularization in PDR, were significantly upregulated under combined DMOG and LPS stimulation compared with monostimulation (Figure 4A–C). Additionally, snail family transcriptional repressor 1 (SNAI1), a master transcription factor that regulates mesenchymal transition, was upregulated under both DMOG and LPS stimulation (Figure 4D). Correspondingly, collagen type I alpha 1 chain (COL1A1), a major extracellular matrix (ECM) component induced by SNAI1 activation, was markedly increased under the combined stimulation (Figure 4E).

These results underscore the synergistic effects of hypoxia and inflammation in activating gene expression pathways that contribute to the pathological angiogenesis and fibrotic characteristics of PDR, further highlighting the pivotal role of microglia in disease progression.

### 2.5. Celecoxib Inhibits COX-2-Mediated Inflammatory, Angiogenic, and Fibrotic Gene Expression in iMGs Under Hypoxia and Inflammation

**Figure 5:**
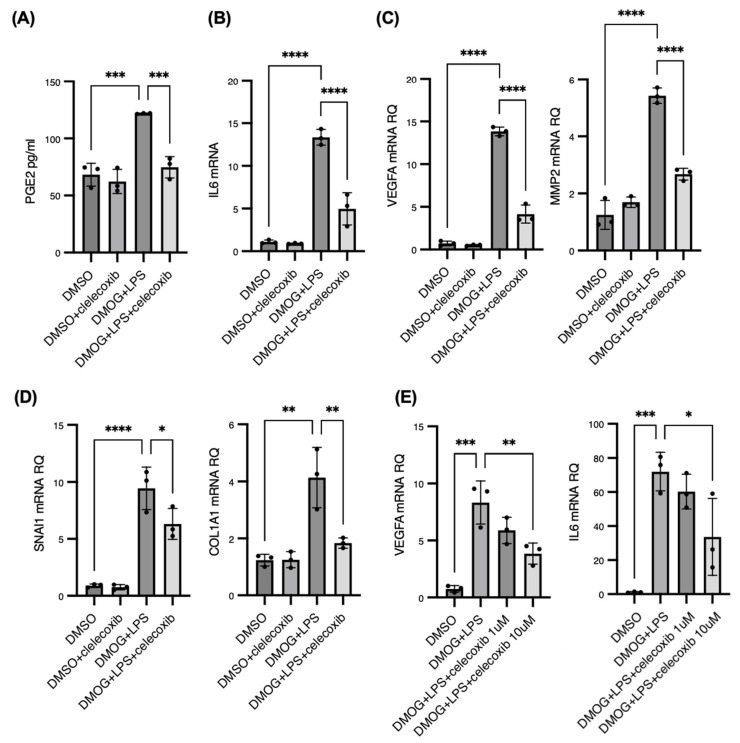
Celecoxib inhibits cyclooxygenase 2 (COX-2) mediated inflammatory, angiogenic, and fibrotic gene expression in induced pluripotent stem cell-derived microglia (iMGs) under hypoxia and inflammation. We treated iMGs stimulated by 200 μM dimethyloxaloylglycine (DMOG) and 100 ng/mL lipopolysaccharide (LPS) with 10 μM celecoxib, the selective COX-2 inhibitor, for 24 h. Celecoxib treatment significantly downregulated prostaglandin E2 production in the culture supernatant (**A**). The quantitative polymerase chain reaction results showed that celecoxib markedly reduced the expression of interleukin-6 (IL-6) in both DMOG and LPS-stimulated iMGs (**B**). Celecoxib significantly decreased the expression of vascular endothelial growth factor A (VEGFA) and matrix metalloproteinase 2, the critical drivers of pathological neovascularization, which were notably upregulated under combined DMOG and LPS stimulation (**C**). The expression of snail family transcriptional repressor 1, a key transcription factor for fibrosis, and its downstream target, collagen type I alpha 1 chain, was significantly reduced by celecoxib treatment (**D**). The expression of VEGFA and IL-6, which was elevated by DMOG + LPS, was suppressed in a dose-dependent manner by celecoxib (**E**). * *p* < 0.05, ** *p* < 0.01, *** *p* < 0.001, **** *p* < 0.0001.

Based on the above results, we hypothesized that COX-2 amplification under hypoxia and innate immune activation in iMGs drives the upregulation of genes associated with angiogenesis and fibrovascular proliferation. To test this hypothesis, we treated DMOG- and LPS-stimulated iMGs with the selective COX-2 inhibitor celecoxib. As expected, celecoxib significantly downregulated PGE2 production under these conditions (Figure 5A).

Celecoxib also markedly reduced the expression of IL-6 in both DMOG- and LPS-stimulated iMGs (Figure 5B). Furthermore, celecoxib significantly decreased the expressions of VEGFA and MMP2, which are critical drivers of pathological neovascularization, and were notably upregulated under combined DMOG and LPS stimulation (Figure 5C). Additionally, the expression of SNAI1, a key transcription factor for fibrosis, and its downstream target, COL1A1, was significantly reduced by celecoxib treatment (Figure 5D). To assess the dose-dependent effects of celecoxib, we evaluated VEGF and IL-6 expression at 1 μM and 10 μM concentrations of celecoxib. The expression of VEGF and IL-6, which was elevated by DMOG + LPS, was not significantly suppressed at 1 μM but was significantly suppressed at 10 μM, indicating a dose-dependent effect (Figure 5E).

These findings confirm our hypothesis that PGE2 mediates the expressions of IL-6, VEGFA, MMP2, and SNAI1, thereby contributing to the inflammatory, angiogenic, and fibrotic phenotypes of activated microglia under hypoxia.

### 2.6. COX-2/PGE2/PGE Receptor 2 Axis as a Central Driver of Angiogenesis and Inflammation in iMGs, with Distinct Pathways Governing Fibrosis

**Figure 6:**
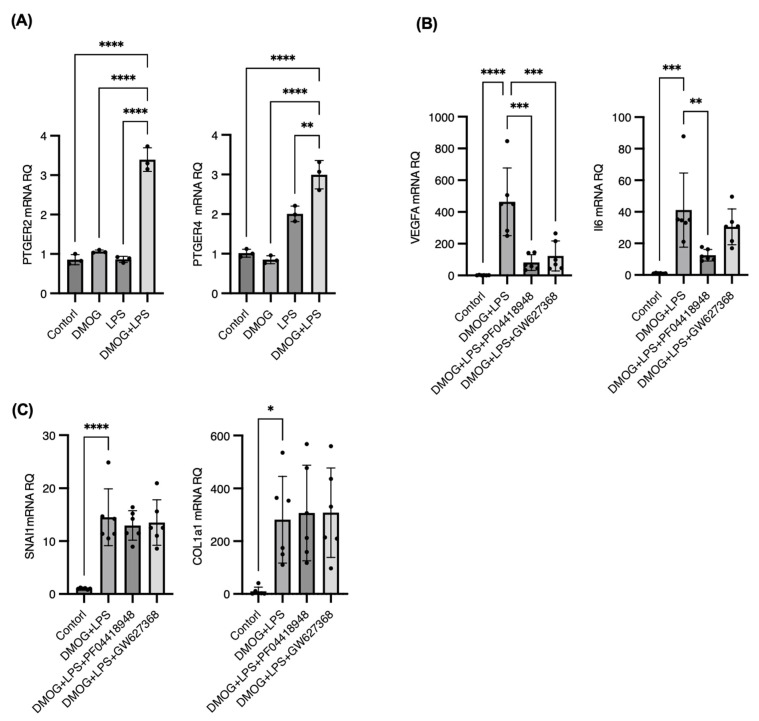
The cyclooxygenase 2/prostaglandin E2/PGE receptor 2 (EP2) axis as a central driver of angiogenesis and inflammation in induced pluripotent stem cell-derived microglia (iMGs), with distinct pathways governing fibrosis. The genes encoding prostaglandin receptors EP2 (PTGER2) and EP4 (PTGER4) were expressed in human iMGs and were notably upregulated by 200 μM dimethyloxaloylglycine (DMOG) and 100 ng/mL lipopolysaccharide (LPS) stimulation for 24 h, whereas the genes encoding EP1 (PTGER1) and EP3 (PTGER3) were not expressed (**A**). To pinpoint which prostaglandin receptors drive the upregulation of angiogenic and fibrogenic genes, we treated iMGs with specific antagonists PF04418948, targeting EP2, and GW627368, targeting EP4 receptors, under combined 200 μM DMOG and 100 ng/mL LPS stimulation for 24 h. Treatment of iMGs with PF04418948 significantly downregulated vascular endothelial growth factor A and interleukin-6 mRNA expression, underscoring the pivotal role of the EP2 receptor in mediating these pathways (**B**). However, SNAI1 and COL1A1 did not respond to EP2 or EP4 antagonists, indicating that they have different regulatory mechanisms (**C**). * *p* < 0.05, ** *p* < 0.01, *** *p* < 0.001, **** *p* < 0.0001.

To characterize the prostaglandin receptors in iMGs through which PGE2 induced by COX-2 exerts its effects under hypoxia and innate inflammation, we first confirmed the expression of prostaglandin receptors (EP1, EP2, EP3, and EP4) in iMGs, given that PGE2 is known to signal through these receptors. The genes encoding EP2 (PTGER2) and EP4 (PTGER4) were expressed in the iMGs and were notably upregulated by DMOG and LPS stimulation, whereas the genes encoding EP1 (PTGER1) and EP3 (PTGER3) were not expressed (Figure 6A).

To identify the prostaglandin that drives the upregulation of angiogenic and fibrogenic genes under combined DMOG and LPS stimulation, we used specific antagonists targeting the EP2 and EP4 receptors. Treatment of iMGs with the EP2 antagonist, PF04418948, significantly downregulated VEGFA and IL-6 mRNA expression, underscoring the pivotal role of the EP2 receptor in mediating these pathways. However, SNAI1 and COL1A1 did not respond to the EP2 or EP4 antagonist, indicating different regulatory mechanisms (Figure 6B,C).

These results establish that the COX-2/PGE2/EP2 axis is a critical regulator of VEGFA and IL-6 expressions in microglia under hypoxic and inflammatory conditions. The upregulation of EP2 suggests that the synergistic upregulation of PTGS2, VEGFA, and IL-6 likely occurs in an autocrine manner in iMGs within this environment. In contrast, the regulation of the fibrotic gene SNAI1 and its downstream target COL1A1, although COX-2-dependent, appears to involve alternative prostaglandin pathways beyond PGE2 or mechanisms independent of EP receptors. Further studies are required to elucidate these regulatory mechanisms.

In summary, our findings demonstrate that hypoxia and innate immune activation induce distinct yet overlapping gene expression changes in human iPSC-derived microglia. The key pathways involved include hypoxia-responsive signaling and immune activation pathways. PTGS2 and its downstream products play a significant role, with potential implications for the pathophysiology of DR. Moreover, the COX-2/PGE2/EP2 axis is a key regulator of hypoxia and inflammation.

## 3. Discussion

This study provides significant insights into the role of microglia in PDR progression, with particular focus on the synergistic effects of hypoxia and innate immune activation on microglial gene expression. Microglia, the resident immune cells of the central nervous system, play a crucial role in regulating retinal angiogenesis under both physiological and pathological conditions, including DR [7]. Physiologically, microglia are located close to blood vessels and are essential for forming neovascularization [11,12,26]. Recent research has highlighted the association between diabetic vascular dysfunction and microglia–endothelial cross-talk and the association between diabetic neurodegeneration and microglia–neuroglia cross-talk. Inflammatory cytokines from microglia are now recognized as pivotal contributors to DR pathogenesis [12,27].

Our findings revealed two main outcomes: first, the synergistic upregulation of COX-2 and its downstream product, PGE2, in human iMGs under combined hypoxic and inflammatory conditions, and second, the critical role of the COX-2/PGE2/EP2 signaling axis in mediating pathological angiogenesis and inflammation in iMGs under these combined conditions. These findings suggest that the interplay between hypoxia and inflammation exacerbates pathological processes associated with PDR. Inflammation exacerbates DR progression [21,22]. The inflammatory response in DR is partly because of significant polarization changes in microglia under ischemic conditions, which drive various pathological processes through the induction of inflammation.

The distinct gene expression profiles observed in the iMGs under different stimulation conditions (LPS, DMOG, and DMOG + LPS) highlight the complex interplay between hypoxia and inflammation during microglial activation. Our study demonstrated that DMOG and LPS co-stimulation resulted in the significant upregulation of 76 genes, among which PTGS2 was the most prominently elevated. This upregulation suggests that COX-2 plays a pivotal role in pathological processes that occur in microglia, including angiogenesis, cell migration, chemotaxis, and fibrosis. These findings are consistent with those of previous studies indicating that COX-2 is a key mediator of inflammation and pathological angiogenesis in various diseases, including DR [24,25,28,29].

COX-2 is the rate-limiting enzyme in the biosynthesis of prostaglandins from arachidonic acid and is constitutively present in the normal retina [30]. The synergistic upregulation of COX-2 and the subsequent increase in PGE2 production in response to combined DMOG and LPS stimulation further emphasize the role of microglia in the inflammatory and angiogenic processes characteristic of PDR. This synergistic upregulation appears to occur through an autocrine mechanism in iMGs, as DMOG and LPS co-stimulation enhance COX-2 and PGE2 production and increase the expression of EP2 and EP4 receptors themselves. This suggests that PGE2 may act on microglia via these upregulated receptors to further amplify inflammatory and angiogenic responses under hypoxic and inflammatory conditions. Our results also indicate that the COX-2/PGE2/EP2 axis is a central driver of VEGFA and IL-6 expression in iMGs under hypoxic and inflammatory conditions. This signaling exacerbates pathological angiogenesis and neurodegeneration in DR through a paracrine effect on endothelial cells and other neuroglia in the retina [12]. The use of specific antagonists targeting EP2 and the COX-2 inhibitor celecoxib significantly downregulates the expression of these key angiogenic and inflammatory factors, highlighting the potential of the COX-2/PGE2/EP2 signaling pathway as a therapeutic target in PDR [31,32]. Elevated PGE2 levels were observed in the aqueous and vitreous humor of patients with PDR, correlating with the in vitro findings and suggesting that PGE2 contributes to the pathological microenvironment in PDR [28]. Certainly, active diabetic fibrovascular membranes abundantly express COX-2 and its metabolic products, correlating with VEGF levels and the number of blood vessels [33].

ECM synthesis and expansion, including collagen production, along with cell proliferation and neovascularization, are key processes in the development and progression of PDR [34,35]. These mechanisms are typically driven by growth factors in response to hypoxia and inflammatory stimuli, leading to the formation of fibrotic tissue on the retinal surface, which generates tractional force [36]. Although the precise cell types responsible for ECM synthesis and the generation of these tractional forces remain unclear, Müller cells are important in providing the mechanical strength necessary for retinal tractional detachment [37]. Notably, our findings reveal that, under hypoxic and inflammatory conditions, microglia enhance the expression of the fibrogenic gene SNAI1 and its downstream gene COL1A1 (type 1 collagen), which is a major component of the ECM in fibrovascular membranes in PDR [38]. SNAI1 drives fibrovascular proliferation [39]. This novel finding indicates that microglia are involved in both angiogenic and fibrogenic responses under these conditions. Supporting this, recent studies using single-cell transcriptome analysis have identified microglia as the major cellular components of fibrovascular membranes in human PDR [13]. Additionally, recent studies have shown that SNAI1 expression in brain microglia plays a significant role in mitigating secondary neuronal damage following central nervous system injury [40]. These findings suggest that, in the context of PDR, microglia may contribute to ECM production through SNAI1 regulation, potentially playing a role in the formation of fibrotic tissue, similar to Müller glia. Further investigation is required to elucidate the role of upregulated SNAI1 expression in microglia under hypoxic and inflammatory conditions.

Our findings also reveal that SNAI1 and COL1A1 expressions are regulated by celecoxib but not by the EP2 and EP4 receptors, indicating that the regulation of these fibrotic genes involves mechanisms independent of the PGE2/EP2/EP4 axis. This suggests that multiple pathways contribute to the complex pathology of PDR and that understanding these pathways is crucial for developing effective treatments. Future studies should focus on elucidating the distinct pathways governing microglial fibrosis in PDR to develop more targeted therapeutic strategies.

The limitation of this study is that DMOG, a HIF-PH inhibitor, and LPS, which broadly increases inflammatory responses, may not fully replicate the environment characteristic of PDR. DR progresses due to chronic inflammation and chronic ischemia, whereas the relatively short stimulation with DMOG+LPS used in this study induces acute inflammatory and ischemic responses, which may differ from the actual environment of diabetic retinopathy. In future research, we plan to explore the effects of not only LPS but also high glucose stimulation and reactive oxygen species, both of which are implicated in the inflammation associated with diabetic retinopathy.

## 4. Materials and Methods

### 4.1. Cell Culture and Differentiation

Human iMG were generated using the protocol described by Haenseler et al. [41]. The human iPS line (201B7) was provided by RIKEN Bio Resource Center (Tsukuba, Japan). hiPSCs were maintained on iMatrix-511 (Takara, Shiga, Japan)-coated plates with mTeSR™1 medium (STEMCELL Technologies, Cologne, Germany). The cells were passaged every 3–4 days at approximately 80% confluence. Briefly, human iPSCs were cultured to form embryoid bodies (EBs) using AggreWell™ 800 plates (STEMCELL Technologies) to form EBs. The iPSCs were harvested as a single cell suspension and seeded into AggreWell™ plates at a concentration of 4 × 10⁶ cells per well. The cells were cultured in TeSR™1 Medium supplemented with ROCK inhibitor (Y-27632) (Abcam, Waltham, MA, USA). EBs were formed and cultured in EB Medium containing BMP-4 (Thermo Fisher Scientific, Waltham, MA, USA), VEGF (Thermo Fisher Scientific, Waltham, MA, USA), and SCF (Miltenyi Biotec, Waltham, MA, USA) for 3 days. The EBs were then harvested and transferred to low-attachment plates, where they were cultured in T175 flasks with Factory Medium containing XVIVO 15 (LONZA, Basel, Switzerland), GlutaMAX (Thermo Fisher Scientific, Waltham, MA, USA), penicillin-streptomycin, 2-mercaptoethanol, macrophage colony-stimulating factor (M-CSF) (Thermo Fisher Scientific, Waltham, MA, USA), and IL-3 (Thermo Fisher Scientific, Waltham, MA, USA) for further differentiation into macrophage precursor cells (MPCs). After 4 weeks, the MPCs emerged in the supernatant. The MPCs were harvested at 6 and 12 weeks. It was previously revealed that their ontogeny is MYB-independent primitive myeloid cells, which is the same ontogeny as microglia [41,42]. MPCs were subsequently differentiated into microglia in microglial medium containing Advanced Dulbecco’s Modified Eagle Medium/F12 (Thermo Fisher Scientific, Waltham, MA, USA), N2 supplement (Thermo Fisher Scientific), GlutaMAX, penicillin-streptomycin, 2-mercaptoethanol, IL-34 (PEPROTECH, Cranbury, NJ, USA), and GM-CSF (PEPROTECH, Cranbury, NJ, USA) for 7 days.

### 4.2. Experimental Conditions

To investigate innate immune activation and hypoxic response, iMGs were subjected to the following conditions: To mimic innate immune activation, the cells were treated with 100 ng/mL LPS (Sigma-Aldrich Inc., St. Louis, MO, USA) for 24 h. To simulate hypoxia, the cells were treated with 200 μM DMOG (Selleck Chemicals LLC, Houston, TX, USA), an HIF prolyl hydroxylase inhibitor, for 24 h. The cells were also treated with both LPS and DMOG to study the synergistic effects of immune activation and hypoxia. The selective COX-2 inhibitor celecoxib (FUJIFILM Wako chemicals, Tokyo, Japan) was used to treat the iMGs at a concentration of 1 μM. Subsequently, 1 μM of EP2 receptor antagonist PF-04418948 (MedChemExpress, Monmouth Junction, NJ, USA) and the EP4 receptor antagonist GW627368 (MedChemExpress) were used to analyze prostaglandin signals.

### 4.3. RNA Isolation and Real-Time Polymerase Chain Reaction

Total RNA was isolated using an RNeasy mini kit (Qiagen, Hilden, Germany) according to the manufacturer’s instructions and reverse transcribed using ReverTra Ace qPCR Master Mix (TOYOBO, Osaka, Japan) for reverse transcription-qPCR. qPCR was performed using Power-up SYBR Green PCR Master Mix (Thermo Fisher Scientific, Waltham, MA, USA) and primers on a QuantStudio5 Real-Time PCR System (Thermo Fisher Scientific, Waltham, MA, USA). β-actin was used as the reference gene for all experiments. Levels of mRNA expression were normalized to those in controls as determined using the comparative CT (ΔΔCT) method. The primer sequences are listed in (Table S1).

### 4.4. RNA Sequence and Transcriptome Analysis

Total RNA was extracted from iMGs using an RNeasy Mini Kit (Qiagen). RNA quality and concentration were assessed using a Bioanalyzer (Agilent Technologies, Santa Clara, CA, USA). RNA sequencing was performed on an Illumina platform. The resulting data were processed and analyzed for differential gene expression using the DESeq2 package in R (iDEP 2.01). Differentially expressed genes were subjected to pathway analysis using the Kyoto Encyclopedia of Genes and Genomes database. GO analysis was performed to categorize the biological processes associated with gene expression changes.

### 4.5. Patients and Samples

All procedures involving patients adhered to the Declaration of Helsinki. Aqueous humor samples were collected from five patients with PDR accompanied by DME (2 males and 3 females; average age, 64.4 ± 6.2 years) and five age-matched patients without any retinal diseases as control (2 males and 3 females; average age, 64.0 ± 10.1 years) for ELISA assay. Vitreous humor samples were also collected from five patients with PDR (4 males and 1 female; average age, 57.6 ± 5.9 years) and five age-matched patients without any retinal diseases as control (3 males and 2 females; average age, 61.0 ± 5.2 years). All samples were immediately frozen and stored at −80 °C until the assays were performed.

### 4.6. Enzyme-Linked Immunosorbent Assay

The concentration of PGE2 in the iMG culture medium supernatant and human aqueous and vitreous humor from PDR and control patients was assessed using a PGE2 ELISA kit (Cayman, Ann Arbor, MI, USA) and PGF2α ELISA kit (Enzo Life Sciences, Farmingdale, NY, USA) according to the manufacturers’ instructions.

### 4.7. Quantification and Statistical Analysis

All data are presented as means ± standard deviations. Significant differences were analyzed using one-way analysis of variance, followed by Tukey–Kramer post hoc analysis for multiple comparisons, using the GraphPad Prism software version 10.0 (San Diego, CA, USA). A *p*-value < 0.05 was considered statistically significant.

### 4.8. Abbreviations

DR: diabetic retinopathy; iPSC: induced pluripotent stem cell; iMGs: induced pluripotent stem cell-derived microglia; DMOG: dimethyloxaloylglycine; LPS: lipopolysaccharide; PTGS2: prostaglandin-endoperoxide synthase 2; COX-2: cyclooxygenase 2; PGE2: prostaglandin E2; EP2: PGE receptor 2; SNAI1: snail family transcriptional repressor 1; COL1A1: collagen type I alpha 1 chain; VEGF: vascular endothelial growth factor; DME: diabetic macular edema; PDR: proliferative diabetic retinopathy; NVU: neurovascular unit; PHDs: prolyl hydroxylase domain enzymes; HIF-1α: hypoxia-inducible factor 1-alpha; TLR4: Toll-like receptor 4; NF: nuclear factor; qPCR: quantitative polymerase chain reaction; ELISA: enzyme-linked immunosorbent assay; PGF2α: prostaglandin F2α; GO: Gene Ontology; IL-6: interleukin-6; MMP2: matrix metalloproteinase 2; ECM: extracellular matrix; EBs: embryoid bodies; M-CSF: macrophage colony-stimulating factor; MPCs: macrophage precursor cells.

## 5. Conclusions

This study investigated the global gene expression and role of microglia in PDR during hypoxia and immune activation, focusing on the COX-2/PGE2/EP2 signaling axis. Our findings revealed that combined hypoxic and inflammatory stimuli led to a significant and synergistic upregulation of PTGS2 (COX-2), resulting in elevated PGE2 levels. This study also showed that celecoxib, a COX-2 inhibitor, effectively reduced the expression of key angiogenic and inflammatory genes (VEGFA, MMP2, IL-6) and fibrogenic genes (SNAI1, COL1A1) that were upregulated by DMOG and LPS co-stimulation. These results highlight the therapeutic potential of targeting this pathway in PDR.

## Data Availability

Data will be made available by the corresponding author upon reasonable request.

## Supplementary Materials

The following supporting information can be downloaded at: https://www.mdpi.com/article/10.3390/ijms252011307/s1.
