# Supplementary material for: Proliferative Diabetic Retinopathy Microenvironment Drives Microglial Polarization and Promotes Angiogenesis and Fibrosis via Cyclooxygenase-2/Prostaglandin E2 Signaling
Source: Int J Mol Sci. 2024 Oct 21;25(20):11307. doi: 10.3390/ijms252011307 (PMC11508523; doi:10.3390/ijms252011307)
Supplement: Supplementary file 1 [file ijms-25-11307-s001.zip › ijms-3234149-supplementary.pdf]

Table S1. Primers used in this study.

**Genes**	**Sequence**
Human PTGS2 Forward	CGGTGAAACTCTGGCTAGACAG
Human PTGS2 Reverse	GCAAACCGTAGATGCTCAGGGA
Human VEGFA Forward	TTGCCTTGCTGCTCTACCTCCA
Human VEGFA Reverse	GATGGCAGTAGCTGCGCTGATA
Human MMP2 Forward	AGCGAGTGGATGCCGCCTTTAA
Human MMP2 Reverse	CATTCCAGGCATCTGCGATGAG
Human IL6 Forward	AGACAGCCACTCACCTCTTCAG
Human IL6 Reverse	TTCTGCCAGTGCCTCTTTGCTG
Human SNAI1 Forward	TGCCCTCAAGATGCACATCCGA
Human SNAI1 Reverse	GGGACAGGAGAAGGGCTTCTC
Human COL1A1 Forward	GATTCCCTGGACCTAAAGGTGC
Human COL1A1 Reverse	AGCCTCTCCATCTTTGCCAGCA
Human PTGER2 Forward	GACCACCTCATTCTCCTGGCTA
Human PTGER2 Reverse	AACCTAAGAGCTTGGAGGTCCC
Human PTGER4 Forward	TACTCATTGCCACCTCCCTGGT
Human PTGER4 Reverse	GACTTCTCGCTCCAAACTTGGC
